# Loss of CEACAM1, a Tumor-Associated Factor, Attenuates Post-infarction Cardiac Remodeling by Inhibiting Apoptosis

**DOI:** 10.1038/srep21972

**Published:** 2016-02-25

**Authors:** Yan Wang, Yanmei Chen, Yi Yan, Xinzhong Li, Guojun Chen, Nvqin He, Shuxin Shen, Gangbin Chen, Chuanxi Zhang, Wangjun Liao, Yulin Liao, Jianping Bin

**Affiliations:** 1 State Key Laboratory of Organ Failure Research, Department of Cardiology, Nanfang Hospital, Southern Medical University, Guangzhou, 510515, China; 2 Department of Oncology, Nanfang Hospital, Southern Medical University, Guangzhou, 510515, China; 3 Department of Cardiology, Henan Provincial People’s Hospital, Zhengzhou University, Zhengzhou, 450003, China

## Abstract

Carcinoembryonic antigen-related cell adhesion molecule1 (CEACAM1) is a tumor-associated factor that is known to be involved in apoptosis, but the role of CEACAM1 in cardiovascular disease is unclear. We aims to investigate whether CEACAM1 influences cardiac remodeling in mice with myocardial infarction (MI) and hypoxia-induced cardiomyocyte injury. Both serum in patients and myocardial CEACAM1 levels in mice were significantly increased in response to MI, while levels were elevated in neonatal rat cardiomyocytes (NRCs) exposed to hypoxia. Eight weeks after MI, a lower mortality rate, improved cardiac function, and less cardiac remodeling in CEACAM1 knock-out (KO) mice than in their wild-type (WT) littermates were observed. Moreover, myocardial expression of mitochondrial Bax, cytosolic cytochrome C, and cleaved caspase-3 was significantly lower in CEACAM1 KO mice than in WT mice. In cultured NRCs exposed to hypoxia, recombinant human CEACAM1 (rhCEACAM1) reduced mitochondrial membrane potential, upregulated mitochondrial Bax, increased cytosolic cytochrome C and cleaved caspase-3, and consequently increased apoptosis. RhCEACAM1 also increased the levels of GRP78 and CHOP in NRCs with hypoxia. All of these effects were abolished by silencing CEACAM1. Our study indicates that CEACAM1 exacerbates hypoxic cardiomyocyte injury and post-infarction cardiac remodeling by enhancing cardiomyocyte mitochondrial dysfunction and endoplasmic reticulum stress-induced apoptosis.

There is growing evidence of direct links between cancer and cardiovascular disease^1^. Elevated morbidity and mortality due to acute myocardial infarction (AMI) have been found in patients with cancer^2^,^3^, while some tumor-specific molecules, such as CA-125, p53, and prostate-specific antigen, are involved in the development of cardiac dysfunction^4^,^5^,^6^. In addition, elevation of prostate-specific antigen is associated with a higher incidence of major adverse cardiac events after MI^7^. Intriguingly, the tumor suppressor p53 has been confirmed to play a critical role in heart failure (HF) induced by MI or pressure overload. Accumulation of p53 may promote myocyte apoptosis, impair cardiac angiogenesis, induce insulin resistance, and consequently lead to myocardial dysfunction or post-MI cardiac rupture^8^,^9^,^10^,^11^,^12^. These reports indicate that some common mechanisms or signaling pathways may be involved in the development of both cancer and cardiovascular disease. Accordingly, identifying the role of tumor-associated factors in cardiovascular disease may be helpful for development of new therapeutic targets.

Carcinoembryonic antigen-related cell adhesion molecule 1 (CEACAM1, also termed CD66a) is a transmembrane protein that has been recognized as an important tumor-associated factor which suppresses or promotes carcinogenesis in a context-dependent manner^13^,^14^, but it remains unknown whether CEACAM1 has any role like p53 in cardiovascular disease. Although it was originally identified as a tumor-associated gene^15^,^16^,^17^, CEACAM1 has been found to promote apoptosis of various cells such as pulmonary and mammary epithelial cells^18^,^19^, oral keratinocytes^20^, cancer cells and Jurkat T cells^21^,^22^. In addition, this pro-apoptotic effect of CEACAM1 has been found to be mediated by the Bax/cytochrome C/caspase-3 pathway^18^,^19^, an intrinsic pathway for apoptosis that is closely associated with mitochondrial function. CEACAM1 activity depends on specific phosphorylation by Calmodulin kinase II^23^, and a recent study demonstrated that CEACAM1 was up-regulated in cardiomyocytes by hypoxia^24^. In addition, endoplasmic reticulum (ER) stress is also closely associated with apoptosis, but it is completely unknown whether CEACAM1 exerts any influences on ER stress^25^,^26^. Considering that mitochondrial dysfunction, Calmodulin kinase II, ER stress and apoptosis are closely associated with myocardial damage and HF^12^,^27^,^28^,^29^, we hypothesized that accumulation of CEACAM1 would enhance myocardial injury and promote cardiac remodeling.

The incidence of MI and post-MI HF continues to increase, thus novel therapeutic strategies are needed. The aim of this study was to identify whether CEACAM1 is a potential therapeutic target in patients with MI and post-MI cardiac remodeling. Accordingly, we first compared the serum level of CEACAM1 between patients with MI and healthy controls, and then we used CEACAM1-deficient mice to evaluate the exact role of this molecule in myocardial injury and cardiac remodeling after MI. Finally, to explore the potential mechanisms, the influence of loss and gain of CEACAM1 function on mitochondrial activity, ER stress and apoptosis were investigated in cultured cardiomyocytes exposed to hypoxia.

## Results

### Serum and myocardial CEACAM1 is increased in response to MI or hypoxia

**Figure 1:**
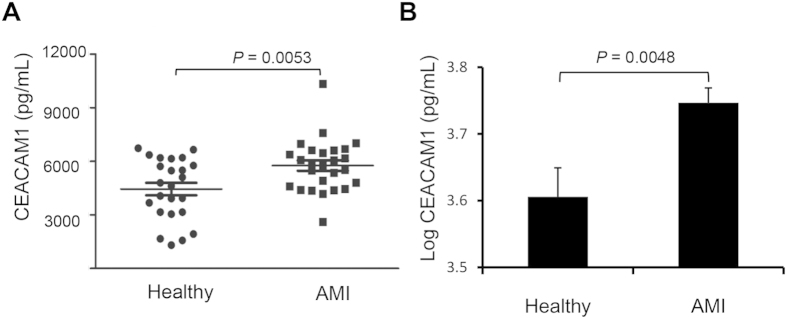
Serum level of CEACAM1 in patients with acute myocardial infarction (AMI). (**A**), Raw data of serum CEACAM1 in patients with AMI (n = 26) and healthy controls (n = 24). (**B**), Logarithmically-transformed serum CEACAM1 data for patients with AMI.

**Figure 2:**
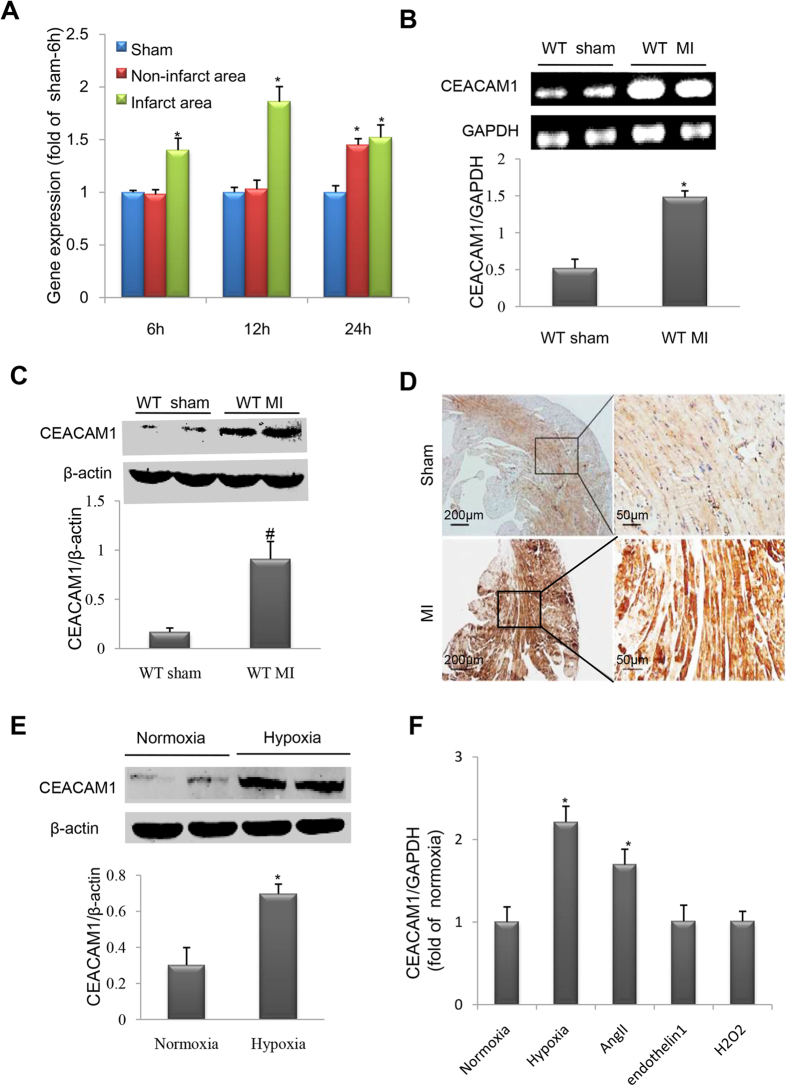
Myocardial CEACAM1 was upregulated in response to myocardial infarction (MI) or hypoxia. (**A**), Time course of CEACAM1 mRNA level in sham, non-infarct area and infarct area. ^*^P < 0.05 vs. sham 6h. Expression of CEACAM1 mRNA (**B**) and protein (**C**) in the left ventricle of wild-type (WT) mice with MI for 3 days (n = 4 in each group). ^*^P < 0.01 vs. sham. (**D**), Immunohistochemical staining shows elevated myocardial CEACAM1 expression in mice with MI than in mice with sham operation (non-infarct area). (**E**), Immunoblotting of CEACAM1 expression in neonatal rat cardiomyocytes exposed to hypoxia for 24 hours (n = 5 in each group). ^*^P < 0.01 vs. normoxia. (**F**), The CEACAM1 mRNA level in neonatal rat cardiomyocytes exposed to hypoxia (for 3h), angiotensin II (AngII, 1 umol/L), endothelin 1(0.1 umol/L) and H_2_O_2_ (0.15 umol/L) for 24 hours. ^*^P < 0.05 vs. normoxia.

Because some circulating tumor-associated factors such as p53 are potential diagnostic and prognostic biomarkers for MI^30^ and HF^31^, we measured the serum level of CEACAM1 in MI patients and healthy controls, revealing that serum CEACAM1 was significantly higher in the MI patients than in the controls (5760 ± 289 pg/mL vs. 4444 ± 350 pg/mL, *P* < 0.01) (Fig. 1). In mice with MI, myocardial CEACAM1 mRNA in the infarct zone started to up-regulate at 6 h, reached the peak at 12 h (Fig. 2A), while in the non-infarct zone it was started to up-regulate at 24 h after MI (Fig. 2A). Eight weeks after MI, myocardial CEACAM1 mRNA and protein expression determined by western blotting and immunohistochemistry in the non-infarct area were significantly higher than in sham group (Fig. 2B–D). In contrast, CEACAM1 was almost undetectable in the fibrosis-formed infarct area at 8 weeks after MI (Supplementary Figure S1A).

Expression of CEACAM1 was also significantly increased in cultured neonatal rat cardiomyocytes (NRCs) exposed to hypoxia (Fig. 2E) but not in cardiac fibroblasts (Supplementary Figure S1B), and CEACAM1 specifically localized on the cell membrane and parts of the cytosol of cardiomyocytes as detected by immunofluorescence (supplementary material Figure S2). In addition, the CEACAM1 expression was significantly increased in cultured NRCs exposed to angiotensin II, but not endothelin1 and H_2_O_2_ (Fig. 2F). These results indicated that CEACAM1 is involved in pathological myocardial stress. Next, we investigated whether loss of CEACAM1 had an influence on cardiac remodeling after MI in mice.

### Loss of CEACAM1 increases post-MI survival

**Figure 3:**
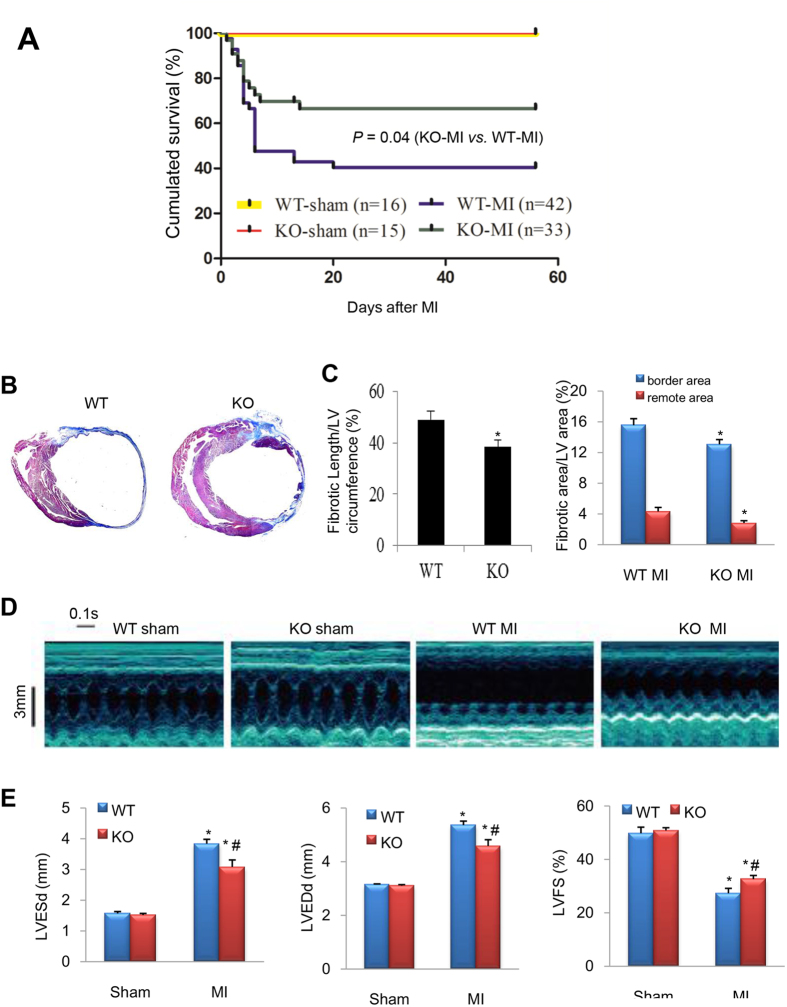
Ablation of CEACAM1 improved the survival rate and post-MI cardiac remodeling. (**A**), Wild-type (WT) and CEACAM1 knockout (KO) mice were subjected to sham operation or MI. Then survival was monitored for 8 weeks. (**B**) Masson trichromatic staining of WT and KO mouse hearts at 8 weeks after MI (blue indicates collagen). (**C**) Quantification of the length of fibrosis in the infarct area (relative to the total LV circumference) and the fibrotic area in the border zone and the remote area. ^*^P < 0.05 vs. the corresponding WT group, n = 6 in each group. (**D**) Representative echocardiographic images from the four groups. (**E**) Quantification of left ventricular end-systolic and end-diastolic diameter (LVESd, LVEDd), and left ventricular fractional shortening (LVFS). ^*^P < 0.01 vs. corresponding sham group, ^#^*P* < 0.05 vs. WT MI. (n = 4 in sham groups; n = 9 in MI groups).

CEACAM1 knockout (KO) mice and their wild-type (WT) littermates were assigned to MI or sham operation. As shown in Fig. 3A, most deaths occurred in the first week due to cardiac rupture or acute heart failure as determined by autopsy. The cumulated survival rate for 8 weeks after MI was only 38.1% in the WT-MI group, while it was 63.6% in the CEACAM1 KO-MI group (*P* < 0.01).

### Reduced post-MI remodeling and cardiac dysfunction in CEACAM1 KO mice

We detected myocardial fibrosis using Masson trichrome staining method, and found that the fibrotic infarct length and fibrosis area in the non-infarct zone were significantly smaller in the CEACAM1 KO-MI group than in the WT-MI group (*P* < 0.05, Fig. 3B,C), suggesting less ventricular expansion in KO mice. Consistent with these findings, echocardiography showed a significant increase of left ventricular end-systolic and diastolic diameters (LVESd and LVEDd), and an obvious decrease of left ventricular fractional shortening (LVFS), in both WT and KO mice with MI compared with the corresponding sham group, while these parameters were comparable between the WT and KO sham groups (*P* < 0.01, Fig. 3D,E). Importantly, the increase of LVESd and LVEDd, and the decrease of LVFS, were smaller in the CEACAM1 KO-MI group than in the WT-MI group (*P* < 0.01, Fig. 3D,E), suggesting that knock-out of CEACAM1 alleviates LV remodeling and dysfunction after MI.

### Deletion of CEACAM1 reduces post-MI apoptosis and downregulates Bax/cytochrome C/caspase -3

**Figure 4:**
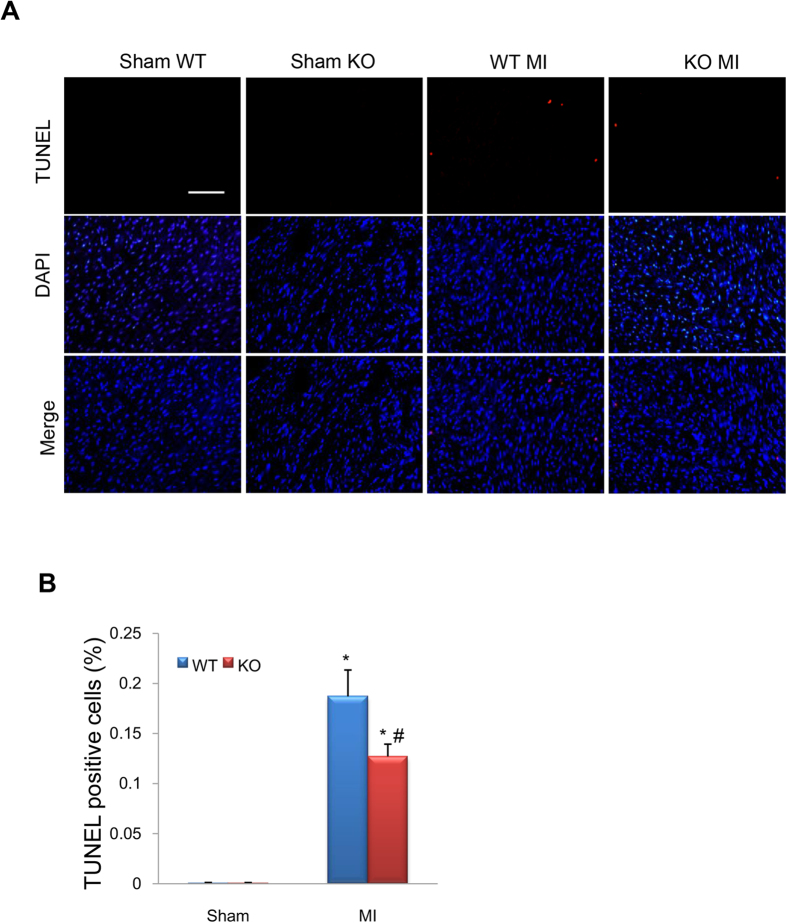
Knockout (KO) of CEACAM1 reduced apoptosis caused by myocardial infarction (MI). TUNEL staining of the posterior wall of the left ventricle (non-infarcted tissue) was performed8 weeks after surgery. (**A**) Representative pictures of TUNEL staining. Scale bar, 50 μm. (**B**) Quantitation of TUNEL-positive cells in the four groups.^*^P < 0.01 vs. wild-type (WT)-sham group, ^#^*P* < 0.05 vs. WT-MI group, n = 8 in each group.

**Figure 5:**
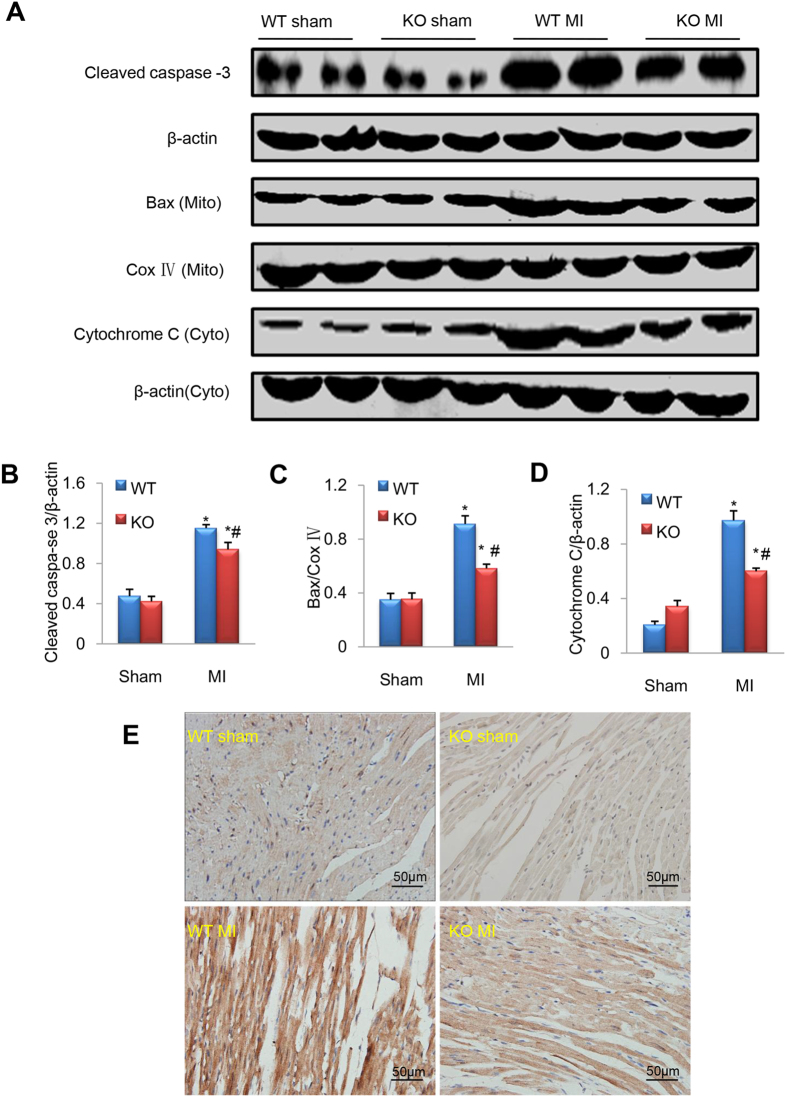
Ablation of CEACAM1 and apoptotic signaling. (**A**) cleaved caspase-3, mitochondrial (Mito) Bax, and cytosolic (Cyto) cytochrome C were analyzed by immunoblotting. CoxIV and β-actin served as loading controls. (**B–D**). Semi-quantitative analysis of cleaved caspase-3 **(B)** mitochondrial Bax **(C)** and cytosolic cytochrome C **(D)**. ^*^P < 0.05 vs. the corresponding sham group; ^#^*P* < 0.05 vs. the WT-MI group, n = 5 in each group. (**E**) Immunohistochemistry of cleaved caspase-3 in the remote non-infarct area in each group. Scale bar, 50 μm.

Eight weeks after MI, the terminal deoxynucleotidyl-transferase–mediated dUTP nick-end labeling (TUNEL) assay showed significantly more apoptotic cells (red) in the non-infarct area in the MI groups than in the corresponding sham groups (*P* < 0.01, Fig. 4A,B), while the number of apoptotic cells was much lower in CEACAM1 KO-MI mice than in WT-MI mice (*P* < 0.01, Fig. 4A,B), indicating that deletion of CEACAM1 reduced apoptosis after MI. Western blotting was performed to detect the expression of mitochondrial Bax, cytosolic cytochrome C, and cleaved caspase-3. All 3 proteins showed substantially higher expression in the MI groups than in sham groups (*P* < 0.01, Fig. 5A–D), while expression of these proteins was significantly lower in the CEACAM1 KO-MI group than in the WT-MI group (*P* < 0.01, Fig. 5A–D). Immunohistochemical staining of cleaved caspase-3 was significantly weaker in the non-infarct area of the KO-MI group than the WT-MI group (Fig. 5E). Next, we used loss and gain of function approaches to examine the cause-effect relation between CEACAM1 and cardiomyocyte injury.

### Recombinant human CEACAM1 (RhCEACAM1) promotes hypoxia-induced apoptosis in cardiomyocytes

**Figure 6:**
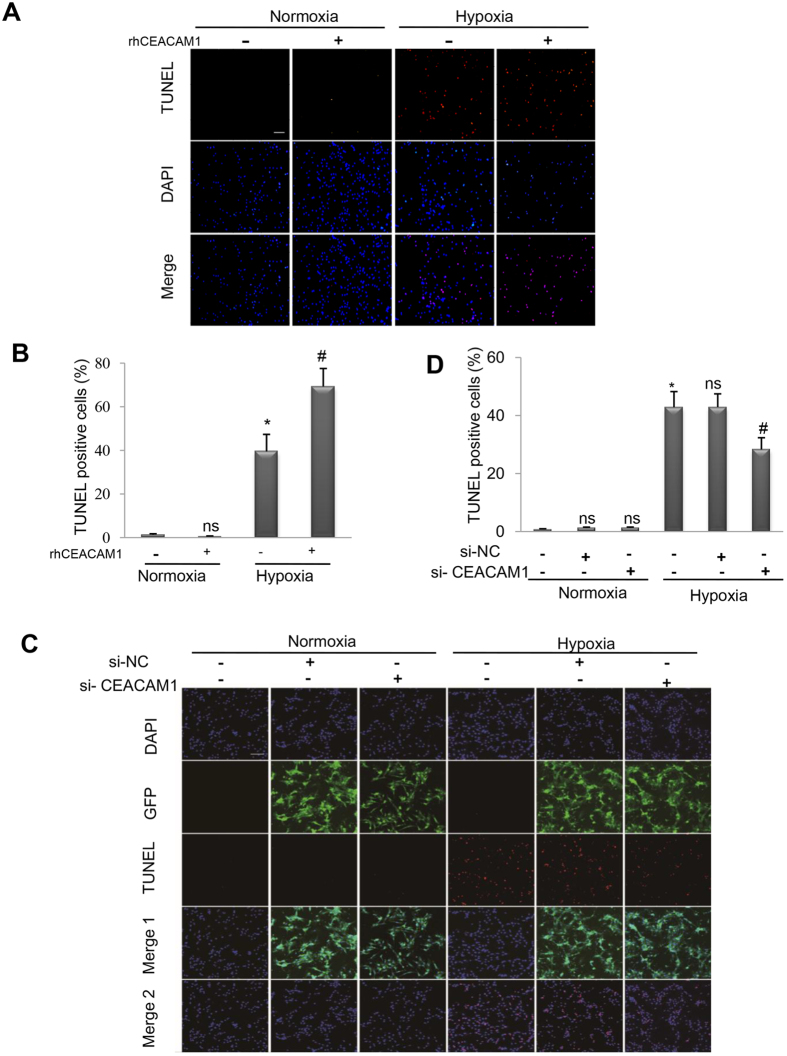
Influence of gain and loss of CEACAM1 function on apoptosis in neonatal rat cardiomyocytes exposed to hypoxia for 24 h. (**A**). TUNEL staining of cardiomyocytes exposed to normoxia or hypoxia in the presence/absence of recombinant human CEACAM1(rhCEACAM1). Scale bar, 50 μm. (**B**), Quantitation of TUNEL-positive cells in the four groups shown in A. ^*^P < 0.01 vs. normoxia, and ^#^*P* < 0.01 vs. hypoxia, n = 6 in each group. (**C**), Representative pictures of TUNEL-stained cultured cardiomyocytes exposed to normoxia or hypoxia in the presence/absence of lentivirus carrying si-RNA for CEACAM1. Scale bar, 20 μm.(**D**), Quantitation of TUNEL-positive cells in the 6 groups shown in C. ^*^P < 0.01 vs. normoxia, ^#^*P* < 0.05 vs. hypoxia +negative control-siRNA (si-NC), n = 10 in each group.

Cultured NRCs were exposed to normoxia or hypoxia in the presence/absence of rhCEACAM1 or lentivirus carrying si-CEACAM1. The TUNEL assay showed that rhCEACAM1 enhanced hypoxia-induced apoptosis, while si-CEACAM1 reduced it (Fig. 6A–D). Next, we investigated how CEACAM1 promoted apoptosis.

### RhCEACAM1 enhances the hypoxia-induced decrease of mitochondrial membrane potential in cardiomyocytes

**Figure 7:**
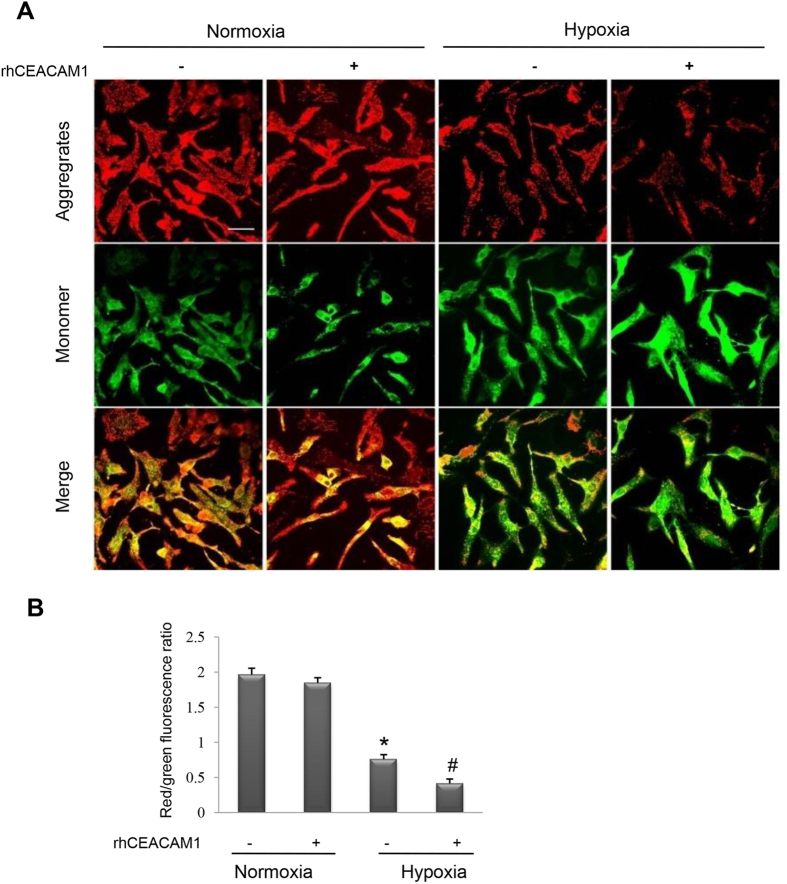
Influence of recombinant human CEACAM1 (rhCEACAM1) on the mitochondrial membrane potential (ΔΨ_m_) in neonatal rat cardiomyocytes exposed to hypoxia for 24 h. JC-1 fluorescent dye in the mitochondrialmatrix produces red fluorescence. If the mitochondrial membrane potential (ΔΨ_m_) declines, JC-1 becomes a monomer in the matrix and produces green fluorescence. (**A**), Representative pictures of JC-1 fluorescence (indicating mitochondrial depolarization) detected by laser confocal microscopy. Scale bar, 20 μm. (**B**), Quantitative analysis of fluorescence intensity in the mitochondria of cardiomyocytes shown in A. RhCEACAM1 decreased the ΔΨ_m_ of hypoxic cardiomyocytes (n = 4 in each group). ^*^P < 0.01 vs. normoxia and ^#^*P* < 0.05 vs. hypoxia.

We observed the effect of rhCEACAM1 on the mitochondrial membrane potential (ΔΨ_m_) by using the lipophilic cationic probe JC-1. When NRCs were exposed to hypoxia, treatment with rhCEACAM1 induced a lower ΔΨ_m_ compared with the control group (*P* < 0.05), while no significant difference was found between rhCEACAM1-treated and rhCEACAM1-untreated groups under normoxia conditions (Fig. 7A,B). These findings suggested that rhCEACAM1 reduces the mitochondrial membrane potential in cardiomyocytes.

### RhCEACAM1 upregulates apoptotic signaling in cardiomyocytes

**Figure 8:**
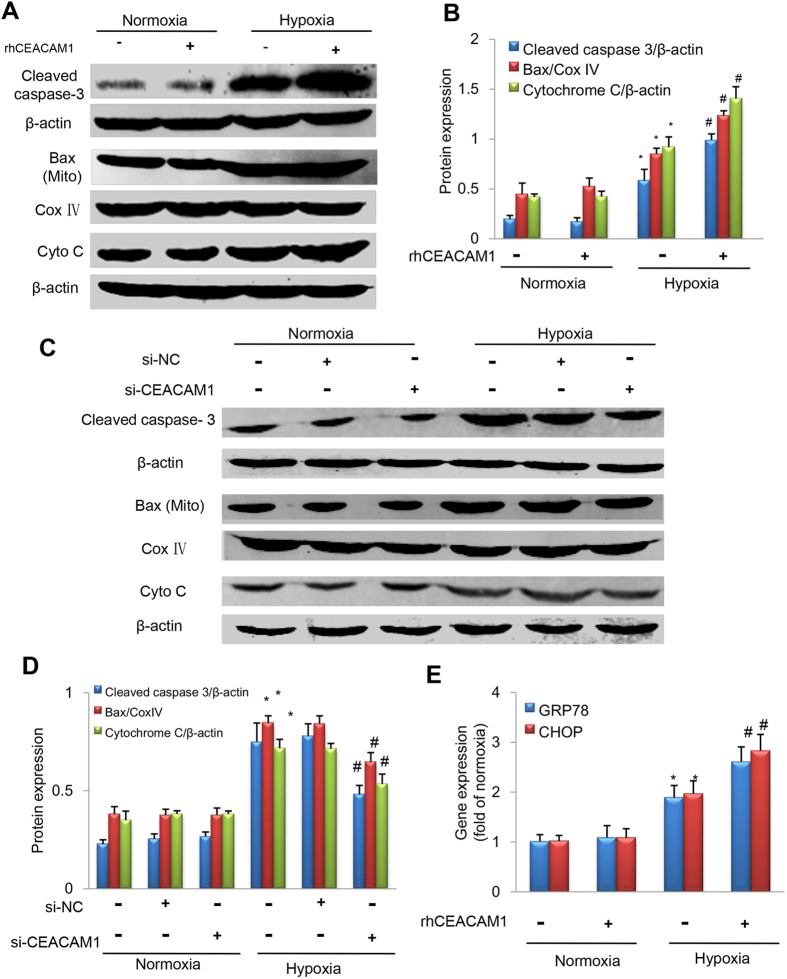
Influence of CEACAM1 on apoptotic signaling pathways in neonatal rat cardiomyocytes exposed to hypoxia for 24 h. Cleaved caspase-3, mitochondrial Bax (Mito Bax), and cytosolic cytochrome C (Cyto C) were analyzed by Western blotting under either normoxic or hypoxic conditions. CoxIV and β-actin served as internal controls. (**A**) Representative western blots of the target and loading control proteins from cardiomyocytes with/without recombinant human CEACAM1 (rhCEACAM1). (**B**) Quantitative analysis of the protein expression shown in A. ^*^P < 0.05 vs. normoxia, ^#^*P* < 0.05 vs. hypoxia, n = 5 in each group. (**C**) Western blotting of cleaved caspase-3, mitochondrial Bax, and cytosolic cytochrome C in cardiomyocytes treated with negative control siRNA (si-NC) or siRNA for CEACAM1 (si-CEACAM1).^*^P < 0.01 vs. normoxia, ^#^*P* < 0.05 vs. hypoxia+ si-NC, n = 5 in each group. (**E**) Quantitative analysis of GRP78 and CHOP mRNA levels from cardiomyocytes with/without recombinant human CEACAM1 (rhCEACAM1). ^*^P < 0.05 vs. normoxia, ^#^*P* < 0.05 vs. hypoxia, n = 5 in each group. (**F**) The GRP78 and CHOP mRNA levels in cardiomyocytes treated with si-NC or si-CEACAM1.^*^P < 0.01 vs. normoxia, ^#^*P* < 0.05 vs. hypoxia+ si-NC, n = 5 in each group.

Mitochondrial Bax, cytosolic cytochrome C, and cleaved caspase-3 are classic markers of apoptosis^27^. Western blotting showed (Fig. 8A) that the expression of these 3 proteins was much higher in the hypoxia group than in the normoxia group (Fig. 8B, *P* < 0.01). Moreover, treating cardiomyocytes with rhCEACAM1 under hypoxic conditions enhanced translocation of Bax to the mitochondria, induced cytochrome C leakage into the cytosol, and promoted activation of caspase-3, as reflected by higher expression of mitochondrial Bax, cytosolic cytochrome, while the upregulation of those 3 proteins was significantly antagonized after transfection with si-CEACAM1 (Fig. 8C,D). These findings suggested that CEACAM1 induces apoptosis during hypoxia through mitochondrial pathway.

### RhCEACAM1 induces cardiomyocyte apoptosis through ER stress pathway

Glucose regulated protein78 (GRP78) and C/EBP homologous protein (CHOP) are classic markers of ER stress. The result of quantitative real-time PCR showed that GRP78 and CHOP mRNA levels were much higher in the hypoxia group than in the normoxia group (Fig. 8E, *P* < 0.05). Moreover, treating cardiomyocytes with rhCEACAM1 under hypoxic conditions increased the level of GRP78 and CHOP (Fig. 8E, *P* < 0.05). Taken together, these findings suggest that ER stress is another pathway for CEACAM1 enhancing cardiomyocyte apoptosis under hypoxia condition.

## Discussion

**Figure 9:**
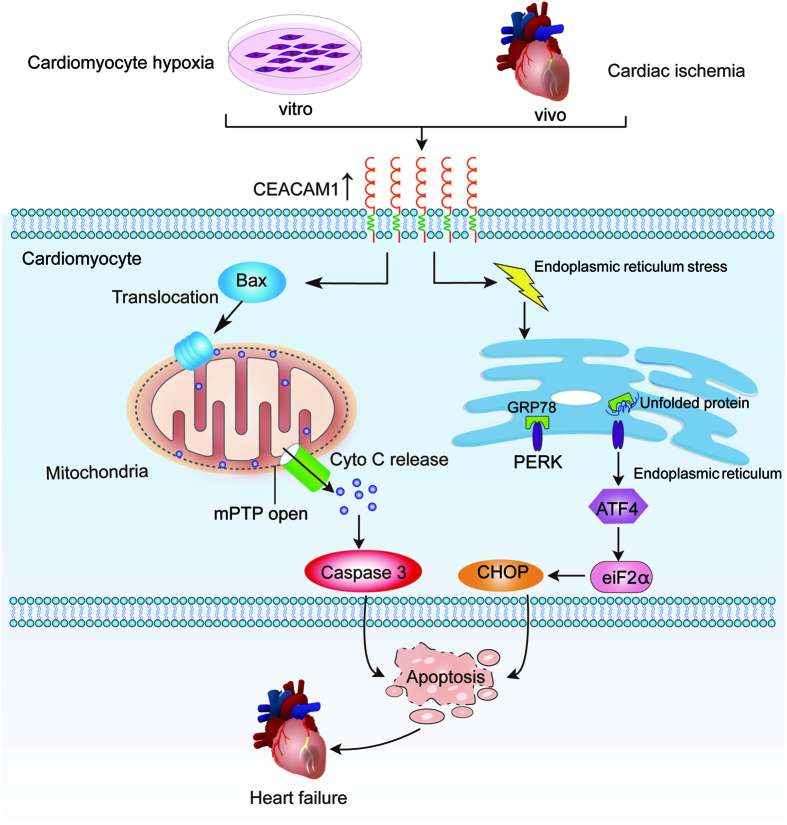
Illustration of the signal pathways of CEACAM1 exacerbateing hypoxic cardiomyocyte injury and post-infarction cardiac remodeling. Both *in vitro* and *in vivo*, CEACAM1 induces mitochondrial translocation of Bax and mitochondria dysfunction with consequent activation of the cytochrome C-caspase-3 apoptotic signaling pathway, which promotes hypoxia-induced apoptosis and of post-infarction cardiac remodeling. In addition, CEACAM1 promotes hypoxia-induced cardiomyocyte apoptosis through GRP78 and CHOP pathway.

The principal findings of the present study were as follows: (1) serum CEACAM1 was elevated in MI patients, and CEACAM1 was increased in the hearts of MI mice and in cardiomyocytes exposed to hypoxic stress. (2) CEACAM1 enhanced hypoxia-induced apoptosis by promoting mitochondrial dysfunction and ER stress. (3) CEACAM1 deficiency contributed to attenuation of cardiac remodeling and dysfunction after MI in mice, resulting in an increase of the survival rate. These findings suggest that CEACAM1, a well-known tumor-specific gene, may play a role in the pathophysiology of MI and cardiomyocyte injury. A summary of the possible mechanisms is shown in Fig. 9.

CEACAM1 is a multifunctional, signal-regulatory protein that is expressed in various cell types, including granulocytes and epithelial cells^32^, while soluble CEACAM1 has been found in body fluids such as saliva, serum, seminal fluid, and bile^17^,^33^,^34^,^35^. However, little is known concerning the expression and role of CEACAM1 in the heart. An increase of CEACAM1transcripts in whole blood was reported in Kawasaki disease^36^, but there have been no investigations into the changes of circulating CEACAM1 in common cardiovascular diseases. This study provided the first evidence that the serum CEACAM1 level is significantly elevated in MI patients, and we also demonstrated that CEACAM1 is expressed by cardiomyocytes and shows upregulation in response to MI or hypoxia. Simeone *et al.*^17^ reported that high level of serum CEACAM1 in patients with pancreatic cancerwas mainly released from tumor cells, suggesting that serum CEACAM1 can come from diseased tissues. Considering that CEACAM1 is a cytoplasmic membrane-associated protein and can release from the cell membrane into serum, it seems reasonable to speculate that high levels of serum CEACAM1 in patients with AMI came from ischemic cardiomyocytes. Furthermore, our animal and cell experiments using gain and loss of function approaches demonstrated that CEACAM1 exacerbates hypoxic cardiomyocyte injury and post-MI remodeling by enhancing apoptosis through promoting mitochondrial dysfunction and ER stress. These findings suggest that CEACAM1 has potential clinical significance as a biomarker of cardiovascular disease and/or therapeutic target that should be carefully investigated in the future.

CEACAM1 was reported to enhance apoptosis in cancer cells via a mitochondrial death pathway involving increased activity of caspases 3, 6, and 9, reduced expression of Bcl-2, mitochondrial translocation of Bax, and an increase of cytosolic apoptosis-inducing factor^18^. These changes also occur in cardiomyocytes subjected to hypoxia or ischemia as we confirmed in this study. Interestingly, our results show that CEACAM1 itself is not sufficient to induce cardiomyocyte apoptosis, but augments apoptotic cell death under stress conditions. The hypoxia or ischemia appears to be a necessary condition for CEACAM1 exerting pro-apoptotic effects, which is a common phenomenon like the action of other tumor-specific molecules such as P53 and CA-125^37^.

Reports from our laboratory and others have demonstrated that a mitochondria-dependent apoptotic pathway plays a central role in cardiomyocyte apoptosis induced by ischemia/hypoxia^28^,^38^,^39^,^40^. In healthy mammalian cells, the majority of Bax is located in the cytosol, but Bax undergoes a conformational shift and becomes associated with the mitochondrial membrane after apoptotic stimulation by hypoxia or ischemia^41^, which is believed to induce opening of the mitochondrial voltage-dependent anion channel with the release of cytochrome C and other pro-apoptotic factors. In the present study, we found that CEACAM1 increased cardiomyocyte apoptosis accompanied by mitochondrial translocation of Bax, leakage of cytochrome C into the cytosol, and activation of caspase-3. Usually, cytochrome C is loosely associated with the inner membrane of the mitochondria and is released during hypoxic stimulation^42^. Since CEACAM1 is a cytoplasmic membrane-associated protein, its deletion may contribute to stabilization of the mitochondrial membrane during ischemic stress and thus may inhibit the release of cytochrome C. Cytoplasmic cytochrome C can initiate other pro-apoptotic signals^43^, including caspase3. Elevation of caspase-3 not only reflects apoptosis^44^, but is also a sign of myocardial infarction^45^. Furthermore, we found that the CEACAM1 also induced apoptosis through ER stress pathway in cardiomyocytes. ER stress is another mechanism of apoptotic cell death. Previous studies have demonstrated that ER stress contributes to cardiomyocyte apoptosis after myocardial infarction through the CHOP signaling pathway^46^,^47^. Here, we firstly demonstrated that CEACAM1 induced cardiomyocyte apoptosis through both ER stress and mitochondrial pathways.

Cardiomyocyte apoptosis plays an important role in cardiac remodeling after MI^12^. It was reported that inhibition of apoptosis by deletion of p53 prevents cardiac rupture^48^. This evidence supports our finding that deletion of CEACAM1 suppressed ischemic or hypoxic cardiomyocyte apoptosis and reduced the mortality rate due to cardiac rupture during the acute phase after MI in mice.

It remains unclear how CEACAM1 drives translocation of Bax to the mitochondria, so this point needs to be investigated in the future. Similar to CEACAM1, p53 was long recognized as a tumor suppressor gene, but it is now believed that p53 also plays an important role in the progression of heart failure. Bax can be upregulated by p53 and is involved in p53-mediated apoptosis^49^. Interestingly, induction of DNA damage by CEACAM1 was reported to depend on regulation of p53^50^, providing an important clue to the possible relationship between p53 and CEACAM1.

In addition to inhibition of apoptosis, other mechanisms may have also contributed to the attenuation of post-MI remodeling in CEACAM1-deficient mice. Permanent occlusion of the left coronary artery for 8 weeks in our mouse model would result in loss of cardiac fibroblasts in the infarct zone, followed by scar formation, so intervention affecting myocardial fibrosis in the remote area would influence post-MI cardiac remodeling. In this study, we noted that loss of CEACAM1 significantly reduced myocardial fibrosis in the non-infarct zone, suggesting that CEACAM1 may influence the development of fibrosis or its degradation. Inhibition of fibrosis in the non-infarct zone would have contributed to LV reducing expansion in CEACAM knockout mice. Coincidently, a previous report showed that release of neutrophil-related matrix metalloproteinase-9 (MMP-9), a collagen-degrading enzyme, was increased in CEACAM1-deficient mice with ischemic stroke^51^, while it has been reported that MMP-2/9 can attenuate post-MI fibrosis by preventing the accumulation of collagen^52^.

The exact role of CEACAM1 is controversial and seems to be context-dependent. Genetic loss of CEACAM1 enhances myeloid tumor growth and angiogenesis^53^, whereas CEACAM1 creates a pro-angiogenic microenvironment to support maturation of ductal mammary adenocarcinoma^54^. Considering that angiogenesis also plays an important role in cardiac remodeling associated with ischemic heart disease, it would be warranted to clarify whether CEACAM1 influences myocardial angiogenesis in an ischemia/reperfusion model.

### Limitation

One limitation in this study is that the TUNEL positive cells may include some non-cardiomyocytes such as cardiac fibroblasts. However our findings that fibroblasts didn’t express CEACAM1 and lack of CEACAM1 inhibited myocardial fibrosis suggest that CEACAM1 does not induce apoptosis of cardiomyocytes. In addition, instead of neonatal rat cardiomyocytes, adult mouse cardiomyocytes would be better for *in vitro* experiments.

## Methods

### Measurement of Serum CEACAM1

Institutional Ethics Committee approval of Nanfang Hospital was obtained for all procedures and experiments in accordance with the principles of the Declaration of Helsinki. Written informed consent was obtained from all patients included in this study. Whole blood samples were obtained from 26 patients with MI (≤6 d) and 24 healthy controls. The characteristics of the patients and controls are shown in supplementary material Table S1. The serum concentration of CEACAM1 was determined by using a DuoSet ELISA Development kit (R&D Systems, Minneapolis, USA) according to the manufacturer’s protocol.

### Mice

CEACAM1 KO mice were provided by Dr Nicole (McGill University, Montreal, Canada) and the details of these mice were described previously^55^. CEACAM1 knockout mice and WT littermates were produced by breeding heterozygous mice and their genotypes were determined by genomic PCR (Supplementary material Figure S3A). Total RNA was extracted from tail tissue with a total RNA isolation system (Omega Bio-Tek, Lilburn, USA). The following primers were used: GAPDH, 5SA). The following primer (sense), 5′-TCCACCACCCTGTTGCTGTA-3imer (antisense), 5′-n CEACAM1(mice), 5′- TACATGAAATYGCACAGTCGC-3′ (sense), 5′- CTGCCCCTGGCGCTTGGAA-3′ (antisense).

### MI model

CEACAM1 KO mice and their littermates (male, age 8–10 w, weight 20–25 g) were used for generation of the MI model. All procedures were performed in accordance with our Institutional Guidelines for Animal Research and the investigation conformed to the National Institutes of Health’s (NIH) Guide for the Care and Use of Laboratory Animals (2011). Mice were anesthetized with a mixture of xylazine (5 mg/kg, ip) and ketamine (100 mg/kg, ip), and left thoracotomy and left coronary artery ligation were performed as described previously^56^. Ischemia was judged from both pallor of the myocardium and ST-segment elevation on the electrocardiogram (Supplementary Figure S3B). After 1 or 3 days or 8 weeks, mice were sacrificed by overdose of pentobarbital sodium (150 mg/kg, ip) or cervical dislocation according to the experimental strategy. Tetrazolium chloride staining was used to confirm the reliability of the MI model (Supplementary material Figure S3C).

### Echocardiography

Cardiac dimensions and function were evaluated by echocardiography with a Sequoia 512 system and a 17L-5 probe (Siemens, Germany). After mice were anaesthetized with 1.5% inhalational isoflurane, two-dimensional parasternal short-axis images of the left ventricle were obtained at the level of the papillary muscles. M-mode echocardiography was performed to measure the LVEDd, LVESd, and LVFS.

### Histological examination

#### Evaluation of infarct size and fibrosis by Masson trichrome staining

Eight weeks after surgery, LV tissues were rapidly excised from mice, rinsed with phosphate-buffered saline, fixed in 4% paraformaldehyde, and embedded in paraffin. Then 4–6 um sections were cut for histological studies. To evaluate the extent of cardiac fibrosis, heart sections were stained with Masson trichrome staining (staining collagen blue and myocardium red). The percent area of cardiac fibrosis was determined from 10 random images obtained in each animal and was calculated as the ratio of the Masson trichrome-stained area to the total optical field using Image J.

#### Determination of cell apoptosis by TUNEL staining

An *In situ* Cell Death Detection kit, TMR red (Roche, Mannheim, Germany) was used to perform TUNEL of myocardial tissues or cultured cardiomyocytes. The TUNEL method was used to label apoptotic cells (red) and DAPI was used to label nuclei (blue). For evaluation of apoptosis *in vivo*, LV sections were treated according to the manufacturer’s instructions, and the slides were covered with 4,6-diamidino-2-phenylindole. The percentage of TUNEL-positive nuclei (red) compared to total nuclei was determined in the myocardium remote from the infarct zone of each animal.

#### Immunohistochemistry

To detect expression of CEACAM1 and cleaved caspase-3 in the myocardium, sections of the middle LV slice were incubated overnight at 4 °C with rabbit anti-CEACAM1 antibody (Santa Cruz Biotechnology, Dallas, USA) or rabbit anti-cleaved caspase-3 antibody (Abcam, Cambridge, UK). Non-immunized rabbit IgG was used as the negative control. Then the sections were incubated with biotinylated anti-rabbit IgG and covered with streptavid in peroxidase, after which peroxidase activity was visualized with 3,3′-diaminobenzidine tetrahydrochloride.

#### Immunofluorescence assay

Cardiomyocytes were plated on fibronectin-precoated chamber slides, post-fixed in Zinc fixative (BD Pharmingen), and blocked for 1 h with diluted normal serum. After incubation overnight with the primary anti-CEACAM1 antibody (Santa Cruz Biotechnology, Dallas, USA), slides were incubated for 1 h at room temperature with the Alexa Fluor 488-conjugated secondary antibody (Invitrogen, Carlsbad, USA). After thorough washing with PBS, the slides were stained with DAPI for10 minutes at room temperature. Following two more washes with PBS, the slides were covered in Immuno-mount (Thermo Scientific, Waltham, USA) and coverslips. An Olympus Fluoview confocal microscope was used to visualize the cells and recorded images were employed for further analysis.

### Cell culture

Cardiomyocytes for *in vitro* experiments were harvested from 1-to 2-day-old Sprague-Dawley rats. The neonatal SD rats were anesthetized by 2% isoflurane inhalation. Isolation and culture of ventricular cardiomyocytes from neonatal rats were performed as described previously^27^. Cells were incubated in Dulbecco’s Modified Eagle’s Medium (DMEM, Invitrogen, Carlsbad, USA) with 10% fetal bovine serum (Invitrogen). Either rhCEACAM1(R&D Systems, Minneapolis, USA) (0.1 μg/mL), or lentivirus carrying si-CEACAM1 were added to mimic or silence CEACAM1 in the cultured cells. In the hypoxia group, serum-free, low-glucose DMEM was pre-equilibrated for 24h in an anaerobic chamber containing a mixture of 1% O_2_, 5% CO_2_, and 93% N_2_.

#### Protocol for rhCEACAM1 treatment

Four groups were used: normoxia, normoxia + rhCEACAM1, hypoxia, and hypoxia + rhCEACAM1.After each treatment, the cells were subjected to western blotting, immunofluorescence, the TUNEL assay, and JC-1 staining.

#### Protocol for si-CEACAM1 treatment

Vectors carrying small interfering RNA targeting CEACAM1 (si-CEACAM1) or the negative control (si-NC) were generated by an external company (Genechem, Shanghai, China). The si-RNA sequences of rat CEACAM1 was as follows: 5′-GCACAGTACTTTTGGCTTA-3′. To generate siRNA duplexes, mixed oligonucleotides were subjected to PCR using the following profile: 95 °C for 30 min, 80 °C for 30 min, 70 °C for 30 min, and room temperature for another 30 min. Double-stranded oligonucleotides were subsequently cloned into the GV118 plasmid. The final titer of lentivirus harvested from 293T cells was 1.5E + 9 TU/ml. Then si-CEACAM1 or the non-targeting control (si-NC) was transfected into neonatal rat cardiomyocytes for 24 h, after which the cells were maintained in normal fresh medium for a further 24 h before experiments and analysis (western blotting and TUNEL analysis).The infection efficiency of si-CEACAM1 and si-NC, as well as the silencing efficiency in cardiomyocytes exposed to normoxia or hypoxia, were evaluated using GFP florescence and western blotting, which confirmed that the silencing efficiency of CEACAM1 was about 85% (supplementary material Figure S4A–C).

### Measurement of the mitochondrial membrane potential

The lipophilic cationic probe JC-1 (Beyotime, Shanghai, China) was employed to measure the mitochondrial membrane potential (ΔΨ_m_) of neonatal rat cardiomyocytes according to the manufacturer’s directions. Briefly, cells were incubated with JC-1 staining solution (5 μg/mL) for 20 minutes at 37 °C and rinsed twice with JC-1 staining buffer. Then the fluorescence intensity of both mitochondrial JC-1 monomers (λ_ex_ 514 nm, λ_em_ 529 nm) and aggregates (λ_ex_ 585 nm, λ_em_ 590 nm) was detected using a monochromatic microplate reader (Safire II, Tecan, Switzerland). The ΔΨ_m_ of cardiomyocytes in each group was calculated as the ratio of red to green fluorescence.

### Isolation of mitochondria from mouse hearts and neonatal rat cardiomyocytes

Mitochondria were isolated from cardiac tissue as described previously^57^. The hearts of mice were rapidly excised and minced on ice, re-suspended in 1 mL of homogenization buffer (10 mmol/L tris, 250 mmol/L sucrose, 1× protease inhibitor, pH 7.5) supplemented with 1 mmol/L EDTA, and homogenized with a glass Dounce homogenizer and Teflon pestle. Homogenates were centrifuged at 1000 *g* for 5 minutes at 4 °C, after which the supernatant was re-centrifuged at 10000 *g* for 15 minutes to pellet the mitochondria, and the pellet was washed twice in homogenization buffer without EDTA. The supernatant was re-centrifuged at 50000 rpm for 1 h and the resulting supernatant was used as the cytosolic fraction.

Mitochondria and cytosolic fractions were harvested from cardiomyocytes using a commercially available cytosol/mitochondria fractionation kit according to the manufacturer’s protocol (Beyotime, Shanghai, China). Cellular proteins were extracted with T-PER (Pierce Biotechnology, Rockford, USA) and centrifuged at 12,000 rpm for 20 min. Protein samples were stored at −80 °C.

### Quantitative real-time PCR analysis

The CEACAM1, CHOP and GRP78 mRNA levels were detected using SYBR Green PCR kit purchased from Takara Biotechnology (Takara, Dalian, China) in LightCycler 480 II (Roche Diagnostics, Basel, Switzerland). The primers were displayed in Table S2. Three sets of reactions were performed per sample, and GAPDH was used as the internal reference. The PCR reaction was performed as follows: 95 °C denaturation for 10 min, 40 cycles of 95 °C for 10 s, 58 °C for 20 s, and 72 °C for 31 s. A melting curve created at the end of the qPCR reactions. The level of mRNA was normalized to GAPDH, and the relative expression of mRNA was calculated using the 2-^ΔΔCt^ method.

### Western blotting

Proteins were obtained from non-infarct LV homogenates or cultured cardiomyocytes as described above. Immunoblotting was performed by using antibodies against CEACAM1 (Santa Cruz Biotechnology, Dallas, USA), cytochrome C (Abcam, Cambridge, UK), cleaved caspase-3 (Abcam, Cambridge, UK), Bax (Santa Cruz Biotechnology), or COXIV(Cell Signaling Technology, Danvers, USA). After blocking the membrane with skim milk for 1 h, incubation with the primary antibodies was done overnight at 4 °C. The secondary antibody was Alexa Fluor 680 (Molecular Probes), in a 1:15000 dilution with NFM for 1 h at room temperature. Proteins were visualized with a LI-COR infrared imager and quantitative densitometric analysis was performed using Odyssey software (version 1.2, LI-COR).

### Statistical analysis

Data are expressed as the mean ± SEM. Differences between two groups were evaluated by the unpaired t-test, while one-way ANOVA with post hoc analysis by Bonferroni’s test was employed for multiple comparisons (Graph Pad Prism Software Inc., San Diego, CA). Survival analysis was performed by the Kaplan-Meier method, and between-group differences of survival were tested by the Gehan-Breslow-Wilcoxon test. For all the tests, a probability value of <0.05 was considered to denote statistical significance.

## Additional Information

**How to cite this article**: Wang, Y. *et al.* Loss of CEACAM1, a Tumor-Associated Factor, Attenuates Post-infarction Cardiac Remodeling by Inhibiting Apoptosis. *Sci. Rep.* **6**, 21972; doi: 10.1038/srep21972 (2016).

## Supplementary Material

Supplementary Information
