# Supplementary material for: Loss of CEACAM1, a Tumor-Associated Factor, Attenuates Post-infarction Cardiac Remodeling by Inhibiting Apoptosis
Source: Sci Rep. 2016 Feb 25;6:21972. doi: 10.1038/srep21972 (PMC4766464; doi:10.1038/srep21972)
Supplement: Supplementary Information [file srep21972-s1.doc]

**Loss of CEACAM1, a Tumor-Associated Factor, Attenuates Post-infarction Cardiac Remodeling by Inhibiting Apoptosis**

Yan Wang, MD; ^1^ Yanmei Chen, MD; ^1^ Yi Yan, MD, PhD; ^1^ Xinzhong Li, MD; ^1^ Guojun Chen, MD; ^1^ Nvqin He, MD; ^1^ Shuxin Shen, MD; ^1,4^ Gangbin Chen, MD; ^1^ Chuanxi Zhang, MD; ^1^ Wangjun Liao, MD, PhD; ^2^ Yulin Liao, MD, PhD;^1*^ and Jianping Bin, MD, PhD^1*^

Table S1. Features of patients with acute myocardial infarction and healthy control subjects

| **parameters** | **Healthy** | **AMI** | **P value** |
| --- | --- | --- | --- |
| Serum CEACAM1 (pg/ml) | 4444±350 | 5760±289 | 0.005 |
| male | 3813±557 | 5664±438 |  |
| female | 5075±361 | 5915±292 |  |
| Gender (male/female) | 1.0 (12/12) | 1.6 (16/10) | 0.412 |
| Age (years) | 37±1.5 | 70±1.9 | 0.000 |
| male | 37±2.4 | 69±2.7 |  |
| female | 37±2.0 | 72±2.3 |  |
| Use of β-blocker (%) | 0 (0/24) | 23.1 (6/26) | 0.023 |
| Use of ACEI (%) | 0 (0/24) | 23.1 (8/26) | 0.004 |

Table S2. Primer sequences

| **Gene** |  | **Primer sequences** |
| --- | --- | --- |
| CEACAM1  (rat) | sense  antisense | 5′-TAGCAGCGCTGGCATACTTC-3′  5′-CCAGATTGTGGCTGGAGGTT-3′ |
| CHOP | sense  antisense | 5′-TCAGATGAAATTGGGGGCAC-3′,  5′-TTTCCTCGTTGAGCCGCTCG-3′ |
| GRP78 | sense  antisense | 5′-CAAGAACCAACTCACGTCCA-3′,  5′-AACCACCTTGAATGGCAAGA-3′; |
| GAPDH | sense  antisense | 5′-AGAAGGCTGGGGCTCATTTG-3′,  5′-AGGGGCCATCCACAGTCTTC-3′ |

A B

3 3 Cardiomyocytes

* Fibroblasts *

expression

expression

2 2

1 1

CEACAM1

CEACAM1

0

Sham Non-infarct area Infarct area

0

Normoxia Hypoxia

**Figure S1.** Relative CEACAM1 expression. (A) CEACAM1 mRNA level in mice at 8 weeks after MI in sham, non-infarct area and infarct area. ^*^*P*< 0.01 vs. sham. (B) CEACAM1 mRNA level in rat cardiomyocytes or cardiac fibroblasts under normoxic or hypoxic conditions.

DAPI

Normoxia

CEACAM1

Merge

Hypoxia


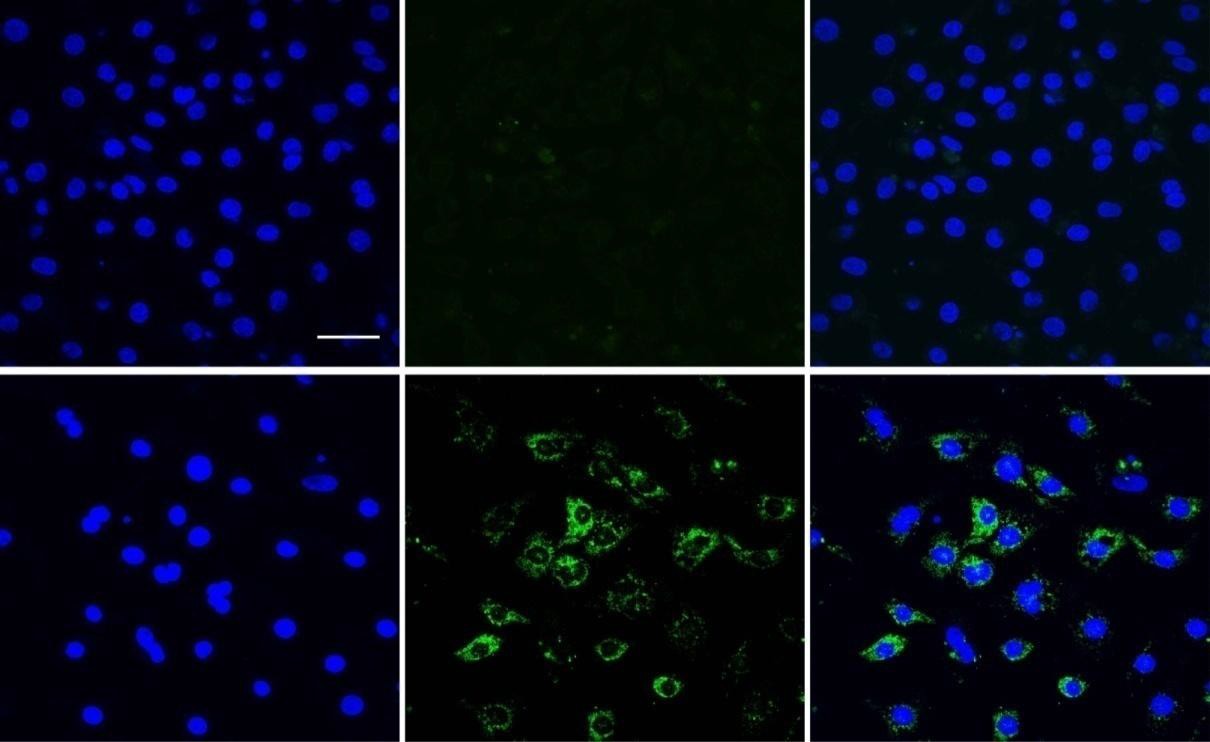


**Figure S2.** Immunofluorescent staining of CEACAM1 in neonatal rat cardiomyocytes under normoxic or hypoxic conditions. Scale bar, 30μm.

**A**

Heterozygous

KO WT

550bp

250bp

**B**


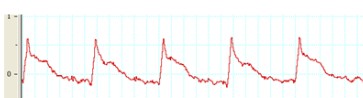


WT

KO
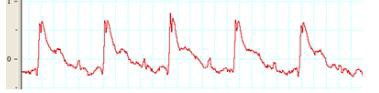


**C**


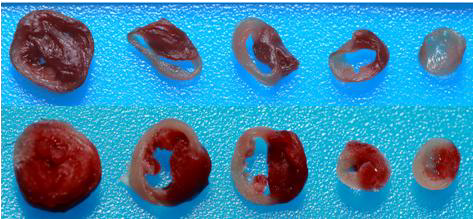


**Figure S3.** Genotyping and confirmation of myocardial infarction model in mice. **(A)** Genotyping of carcinoembyonic antigen-related cell adhesion molecule 1 (CEACAM1) knockout (KO), wild type (WT) and heterozygous mice using PCR. (B) Confirmation of ST segment elevation in ECG in response to coronary ligation. **(C)** Tetrazolium chloride (TTC) stained slices (white indicates dead myocardium, red indicates viable myocardium) from two hearts subjected to ischemia for 24 hours.

**A**

MOI=1

MOI=5

MOI=10

NC siRNA CEACAM1 siRNA


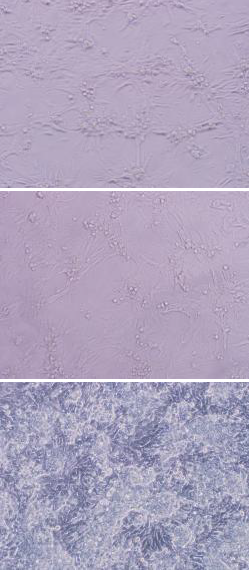


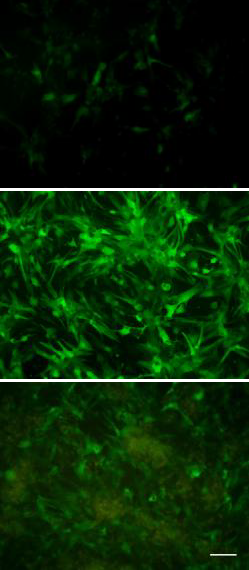


**B**

Hypoxia **- + + +** NC siRNA **- - + -** CEACAM1 siRNA **- - - +**

Ceacam1


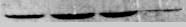


β-actin
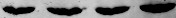


1

0.8 *

Ceacam1/β-actin

0.6

0.4

0.2 #

0

**Figure S4. (A)** Infective efficiency of lentivirus carrying CEACAM1-siRNA or negative control (si-NC) in cultured neonatal rat cardiomyocytes detected by the green fluorescence of co-expressed EGFP. **(B)** Western blot analysis of CEACAM1 levels in response to si-CEACAM1 or si-NC infection in cardiomyocytes expose to hypoxia. #*P*< 0.01 vs. si-NC group, n = 5, ^*^*P*< 0.01vs. nomorxia group. MOI (multiplicity of infection) = 5. Data are mean ± SEM.
